# Prognostic Value of Circulating Tumor Cells in Ovarian Cancer: A Meta-Analysis

**DOI:** 10.1371/journal.pone.0130873

**Published:** 2015-06-22

**Authors:** Yunlan Zhou, Bingxian Bian, Xiangliang Yuan, Guohua Xie, Yanhui Ma, Lisong Shen

**Affiliations:** Department of Clinical Laboratory, Xin Hua Hospital, Shanghai Jiao Tong University School of Medicine, Shanghai 200092, China; The Ohio State University, UNITED STATES

## Abstract

**Background:**

The prognostic value of circulating tumor cells (CTCs) in ovarian cancer has been investigated in previous studies, but the results are controversial. Therefore we performed a meta-analysis to systematically review these data and evaluate the value of CTCs in ovarian cancer.

**Materials and Methods:**

A literary search for relevant studies was performed on Embase, Medline and Web of Science databases. Then pooled hazard ratios (HRs) for survival with 95% confidence intervals (CIs), subgroup analyses, sensitivity analyses, meta-regression analyses and publication bias were conducted.

**Results:**

This meta-analysis is based on 11 publications and comprises a total of 1129 patients. The prognostic value of the CTC status was significant in overall survival (OS) (HR, 1.61;95% CI,1.22–2.13) and progression-free survival (PFS)/disease-free survival (DFS) (HR, 1.44; 95%CI, 1.18–1.75). Furthermore, subgroup analysis revealed that the value of CTC status in OS was significant in "RT-PCR" subgroup (HR, 2.02; 95% CI, 1.34–3.03), whereas it was not significant in "CellSearch" subgroup (HR, 1.15; 95% CI 0.45–2.92) and "other ICC" subgroup (HR, 1.09; 95% CI 0.62–1.90). The presence of CTC was also associated with an increased CA-125 (OR, 4.07; 95%CI, 1.87–8.85).

**Conclusion:**

Our study demonstrates that CTC status is associated with OS and PFS/DFS in ovarian cancer.

## Introduction

Ovarian cancer is the second leading cause of death among gynecologic malignancies in the world [1] owing to the fact that a majority of patients are diagnosed in late stages of the disease [2]. In such a setting, identifying prognostic indicators for patients with ovarian cancer is crucial. CA-125 is a frequently used biomarker in ovarian cancer, but some non-malignant conditions also cause elevated serum CA-125 concentrations [3]. Thus additional prognostic markers are urgently needed for ovarian cancer.

CTCs are tumor cells that have shed into bloodstream from the primary tumors, recurrences, or metastases, and possess antigenic and genetic tumor-specific characteristics. Two major detection methods have been used to identify CTC, including immunocytochemistry (ICC), reverse-transcriptase polymerase chain reaction (RT-PCR). US Food and Drug Administration (FDA) only approved the CellSearch system for clinical use currently, which enriches and detects CTC of epithelial origin by means of ICC methods. There is no published polymorphism studies associated with CTCs in ovarian cancer in previous studies.

CTCs have been demonstrated to have prognostic value among patients with breast, colorectal, gastric, lung and pancreatic cancers in previous meta-analyses [4–8]. However, the value of CTCs in ovarian cancer still remains controversial. Some studies did not observe any correlation between CTC status and prognosis. In contrast, other studies revealed association between CTC status and prognosis. After considering the conflicting results from previous studies, we performed the first meta-analysis to investigate the prognostic value of CTCs on OS and DFS/PFS in patients with ovarian cancer confirmed by histopathologic examinations. Furthermore, subgroup analyses were conducted to evaluate whether the detection method and treatment methods influence the prognostic value of CTCs.

## Methods

### Search strategy

A literary search for potential studies was performed on Embase (from 1974 to November 1, 2014), Medline (from 1966 to November 1, 2014) and Web of Science databases including Science Citation Index Expanded, Social Sciences Citation Index, Arts & Humanities Citation Index, Conference Proceedings Citation Index—Science, Conference Proceedings Citation Index—Social Science & Humanities, Current Chemical Reactions (from 1985 to November 1, 2014). Search term combinations were”ovarian cancer,”“ovary cancer,”“ovarian carcinoma,”“ovary carcinoma,”“ovarium carcinoma,”“circulating tumor cells,”“circulating tumor cell,”“circulating cancer cells,”“circulating cancer cell,”“CTCs” and”CTC” in title/abstract. Relevant articles were also screened manually to prevent omission of any research. If the data in studies were insufficient, we contacted authors by e-mail. And we subsequently excluded the studies when authors couldn’t be contacted.

### Selection criteria and quality assessment

Studies were selected from initial search using following inclusion criteria: (1) survival data were analyzed for the prognostic value of CTCs in ovarian cancer; (2) sufficient data were provided to determine HR with 95% confidence interval (CI); (3) when the same study population was published at several reports, only the most complete one was selected for our meta-analysis; (4) more than 30 patients were enrolled in each study; (5) reports in English were eligible. Studies with reviews, letters, editorials, abstracts, comments and case reports were also excluded.

Two investigators independently evaluated the quality of included studies using the Newcastle-Ottawa Scale (NOS). A score of zero points meant the study had the worst quality, whereas a score of nine points meant the study had the best quality. When disagreements occured, they were solved by discussion. We also conducted subgroup and sensitivity analyses to assess study quality.

### Data extraction

Following details were extracted from included study: name of first author, year of publication, patients’ country, number of patients, disease stage, CTC detection methods, target antigen/target gene, cutoff defining positivity of CTC, HRs with 95% CIs for PFS, DFS, OS. HRs and 95% CIs were extracted from multivariable analyses. When they were not directly extracted from the original study, they were calculated by the method of Tierney et al.[9]. When more than 1 blood sample per patient was detected at different time points such as baseline, mid-therapy and post-therapy, we only investigated baseline value of CTC in ovarian cancer.

### Statistical analysis

As the detection methods of CTCs, and detection rates of CTC in patients were very different across studies, a random effect model was used for calculating the pooled HR [10,11]. In order to evaluate potential sources of heterogeneity, subgroup analyses were performed by detection methods and treatment methods. Stratified analysis with respect to population was not conducted because most of the included studies were from Caucasian countries. Meta-regression analyses were also used to evaluate potential causes of heterogeneity (a p-value<0.05 was considered statistically significant). To evaluate the influence of single studies on the pooled HRs, we performed a sensitivity analysis by estimating the average HR in the absence of each study. To investigate whether a publication bias might have affected the validity of the estimates [12], we performed Begg’s funnel plot and Egger’s linear regression test (a p-value<0.05 was considered statistically significant). STATA version 12.0 was performed to process all of the data.

## Results

### Characteristics of identified studies

**Fig 1:**
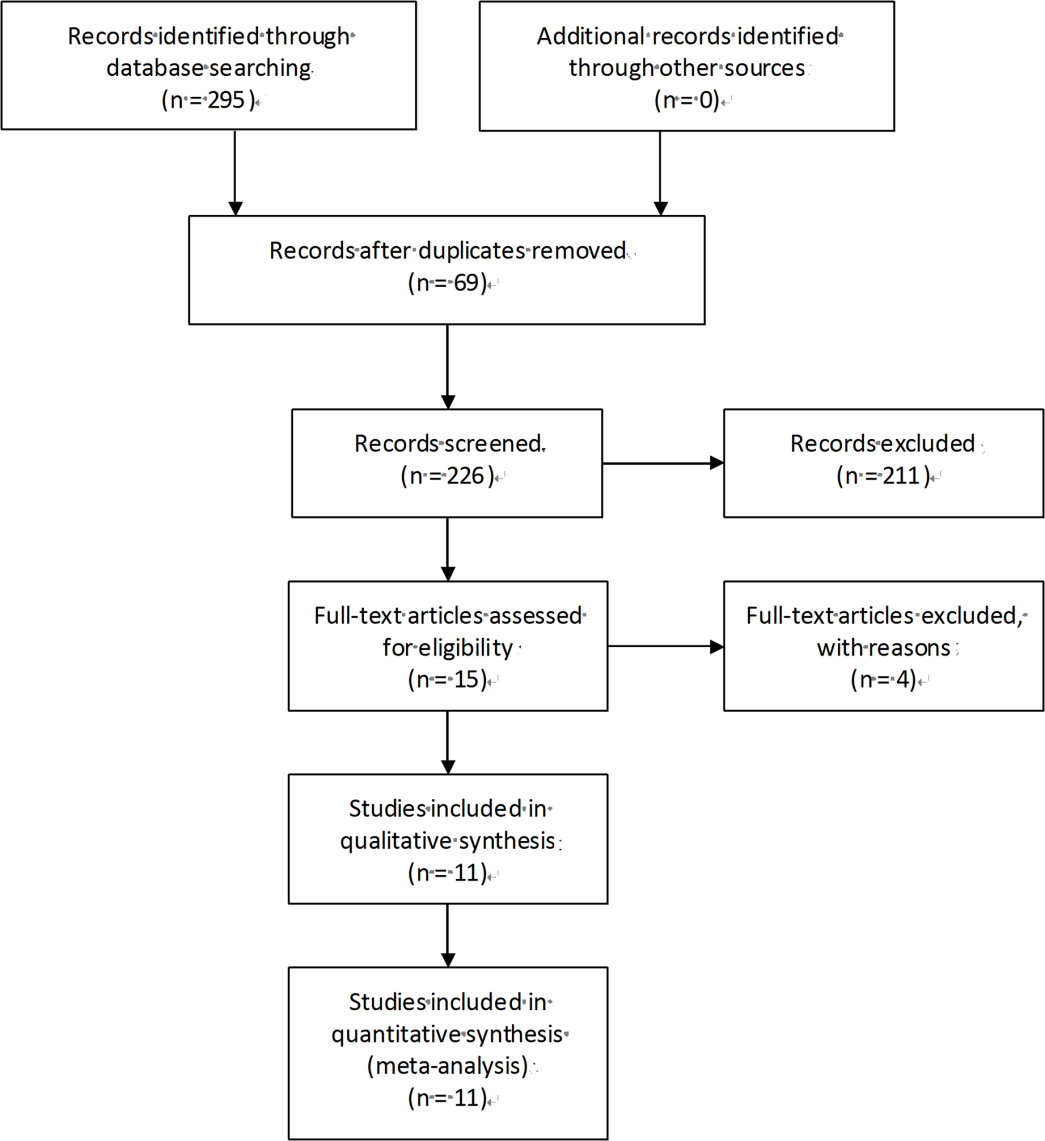
PRISMA flow Chart of literature search and study selection.

The study followed the criterions for systematic review and meta-analysis of genetic association studies (S1 and S2 Checklists). The flowchart of search strategy for articles is presented in Fig 1 and S1 Table. 295 articles related to the keywords were initially reviewed. Of these articles, 280 were excluded after screening of titles, keywords and abstracts because they were obviously irrelevant studies, duplicates, reviews, abstracts and comments. Another 4 articles were excluded after reviewing the full texts because of insufficient data, multiple publications and small sample size, leaving 11 eligible studies [13–23]. published between 2002 and 2014. As shown in Table 1, a total of 1129 patients (ranging from 30 to 216 for individual study) were included in our meta-analysis. The detection rate of CTCs in these patients ranged from 12% to 83%. Most of studies were from Caucasian countries [13–16,18,20–23]. One study was from Asian countries [17] and the other one was from multi-country[19]. Methods used to detect CTCs were CellSearch system, other ICC and the RT-PCR. 64 women with cancer were included in Judson’s study [15], but only 51 patients with new diagnosis were included in the Kaplan–Meier distributions. 60 patients were enrolled in Behbakh’s study, whereas only 43 patients were included in the Kaplan–Meier PFS distributions for pre-treatment CTCs [14]. HRs with 95% CIs were directly extracted from original articles in three studies [17,19,23]. HRs with 95% CIs were not directly reported in eight studies [13–16,18,20–22] and calculated from Kaplan–Meier curves suggested by Tierney et al.[9]. Quality assessment of included studies is shown in Table 2.

**Table 1:** Main characteristics of the eligible studies.

Study	No. of patients	Tumor stage^a^	Detection Method	Target antigen/target gene	Detection rate,%	Cutoff of CTCs
Marth 2002[13] (Norway)	90	Ⅰ-Ⅳ (77.8)	Other ICC	Pancarcinoma/epithelial glycoprotein	12%	—
Judson 2003[15] (America)	51	Ⅰ-Ⅳ (NR)	Other ICC	Keratin 8 and 18,TFS-2,CK-7,CK-20,EGFR	21.6%	—
Fan 2009[20] (America)	66	Ⅰ-Ⅳ (78.8)	Other ICC	CAM,Epi	34.8%	1 CTC/3mL
Poveda 2011[19] (Multi-country)	216	NR (NR)	Cellsearch	CK,EpCAM	14.4%	2 CTC/7.5 mL
Behbakht 2011[14] (America)	43	NR (NR)	Cellsearch	CK,EpCAM	44%	2 CTC/7.5 mL
Aktas 2011[21] (Germany)	122	Ⅰ-Ⅳ (NR)	RT-PCR	EpCAM, MUC-1, HER2, CA 125	19%	—
Obermayr 2013[22] (Europe)	200	Ⅱ-Ⅳ (96)	RT-PCR	PPIC	17%	—
Liu 2013[16] (America)	30	Ⅰ-Ⅳ (100)	Cellsearch	CK,EpCAM	60%	2 CTC/7.5 mL
Sang 2014[17] (China)	80	Ⅰ-Ⅳ (88.8)	RT-PCR	MAGE-A	47.5%	—
Pearl 2014[18] (America)	88	Ⅰ-Ⅳ (80.7)	Other ICC	Epi,HL	83.0%	5 CTC/1mL
Kuhlmann 2014[23] (Germany)	143	Ⅰ-Ⅲ (52)	RT-PCR	EpCAM, MUC1, MUC16	14%	—

^a^Tumor stage and percentage of advanced stage(%); NR, Not Reported; CTCs, circulating tumor cells; ICC, immunocytochemistry; RT-PCR, reverse-transcriptase polymerase chain reaction.

**Table 2:** Main results.

Author	Outcome	HR	95%CI	NOS score
Marth 2002	OS	1.99	0.23–17.16	6
	PFS	2.19	0.84–5.74	
Judson 2003	OS	2.14	0.37–12.29	7
	PFS	1.45	0.55–3.83	
Fan 2009	OS	0.89	0.40–1.95	7
	DFS	1.44	0.78–2.64	
Poveda 2011	OS	1.54	0.93–2.54	7
	PFS	1.58	0.99–2.53	
Behbakht 2011	PFS	1.61	0.79–3.29	6
Aktas 2011	OS	4.56	1.94–10.73	6
	PFS	1.58	0.86–2.88	
Obermayr 2013	OS	2.04	1.17–3.54	7
	DFS	1.35	0.90–2.03	
Liu 2013	OS	0.53	0.12–2.40	7
	PFS	0.71	0.30–1.69	
Sang 2014	OS	1.40	0.87–2.27	6
Pearl 2014	OS	1.06	0.41–2.73	6
	PFS	1.21	0.49–2.97	
Kuhlmann 2014	OS	1.85	1.03–3.32	7
	PFS	1.5	0.81–2.79	

HR, hazard ratio; CI, confidence intervals; NOS, Newcastle-Ottawa Scale; OS, overall survival; PFS,progression-free survival; DFS,disease-free survival.

### CTC and OS

#### Pooled HR

**Fig 2:**
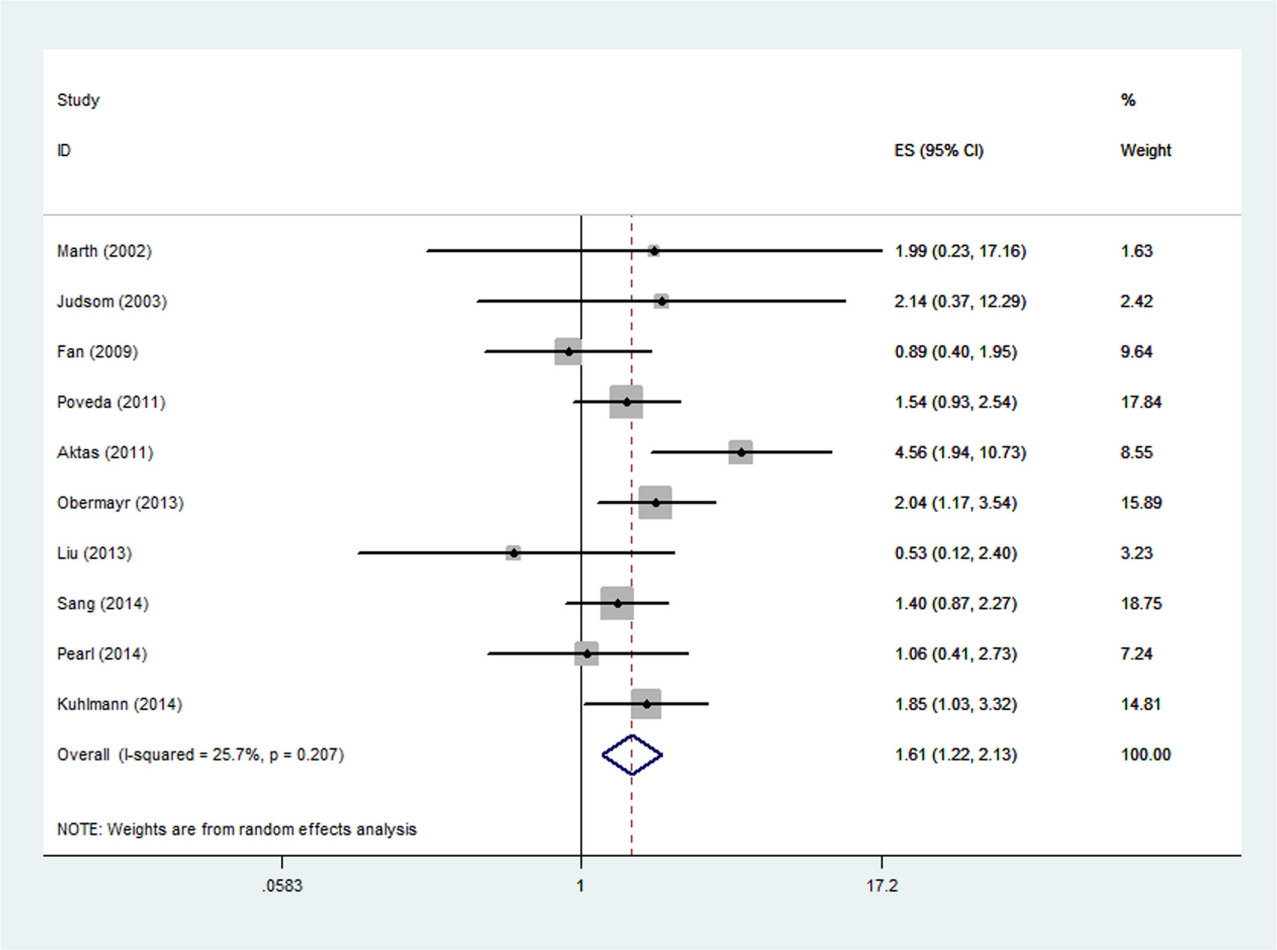
Forest plot showing the meta-analysis of hazard ratio estimates for OS in overall patients. OS = overall survival.

HRs for PFS/DFS were available in ten studies[13,15–23]. The pooled HR showed a significantly increased risk of mortality in patients with CTC positive group (HR, 1.61; 95% CI,1.22–2.13; Fig 2). Heterogeneity among studies was not noted (P = 0.207 and I^2^ = 25.7%).

#### Subgroup analyses

**Fig 3:**
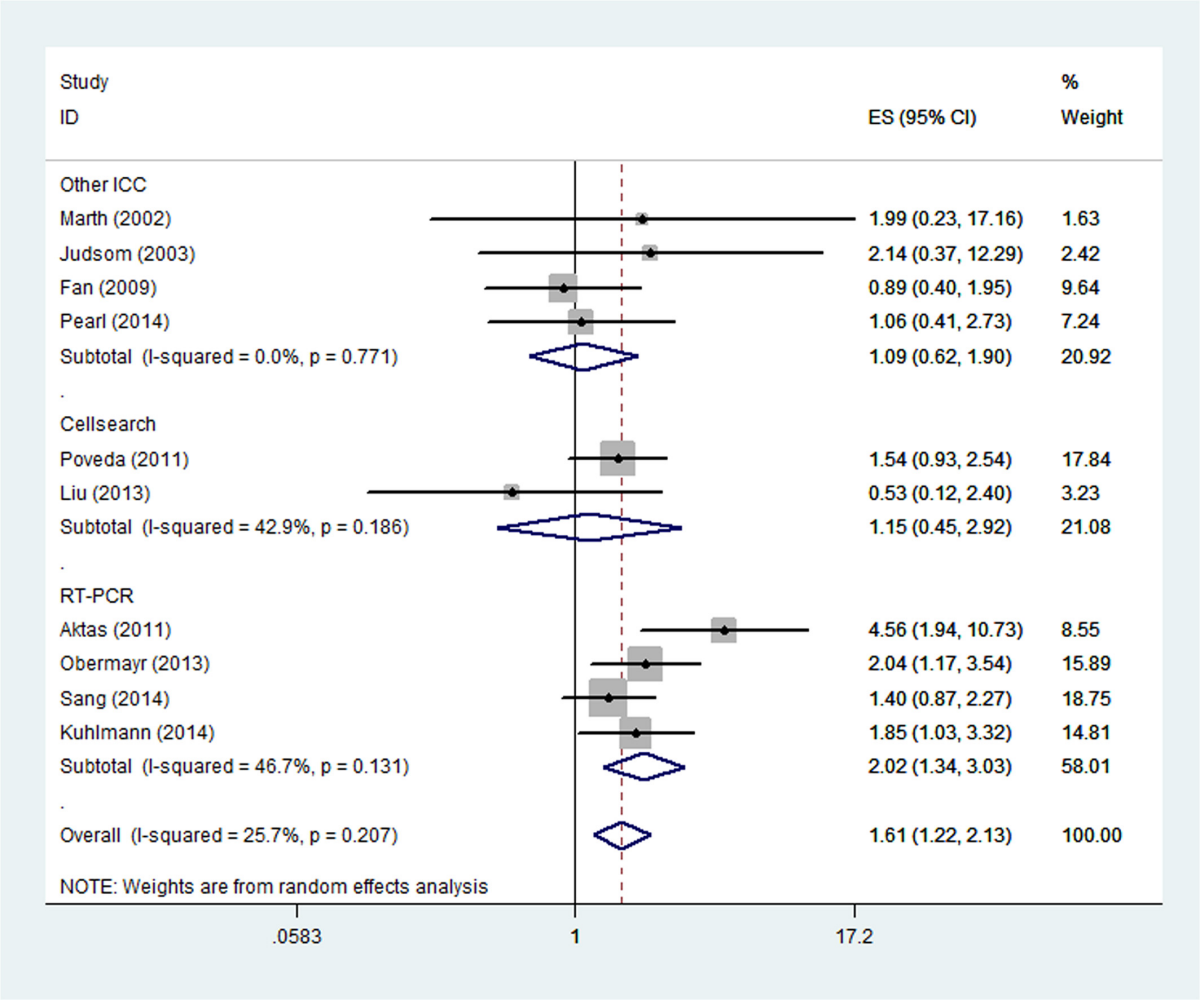
Forest plot showing the meta-analysis of hazard ratio estimates for OS in “Other ICC” subgroup, “Cellsearch” subgroup and “RT-PCR” subgroup. Subgroup analysis based on different CTC detection methods. OS = overall survival; ICC = immunocytochemistry; RT-PCR = reverse-transcriptase polymerase chain reaction; CTC = circulating tumor cell.

**Fig 4:**
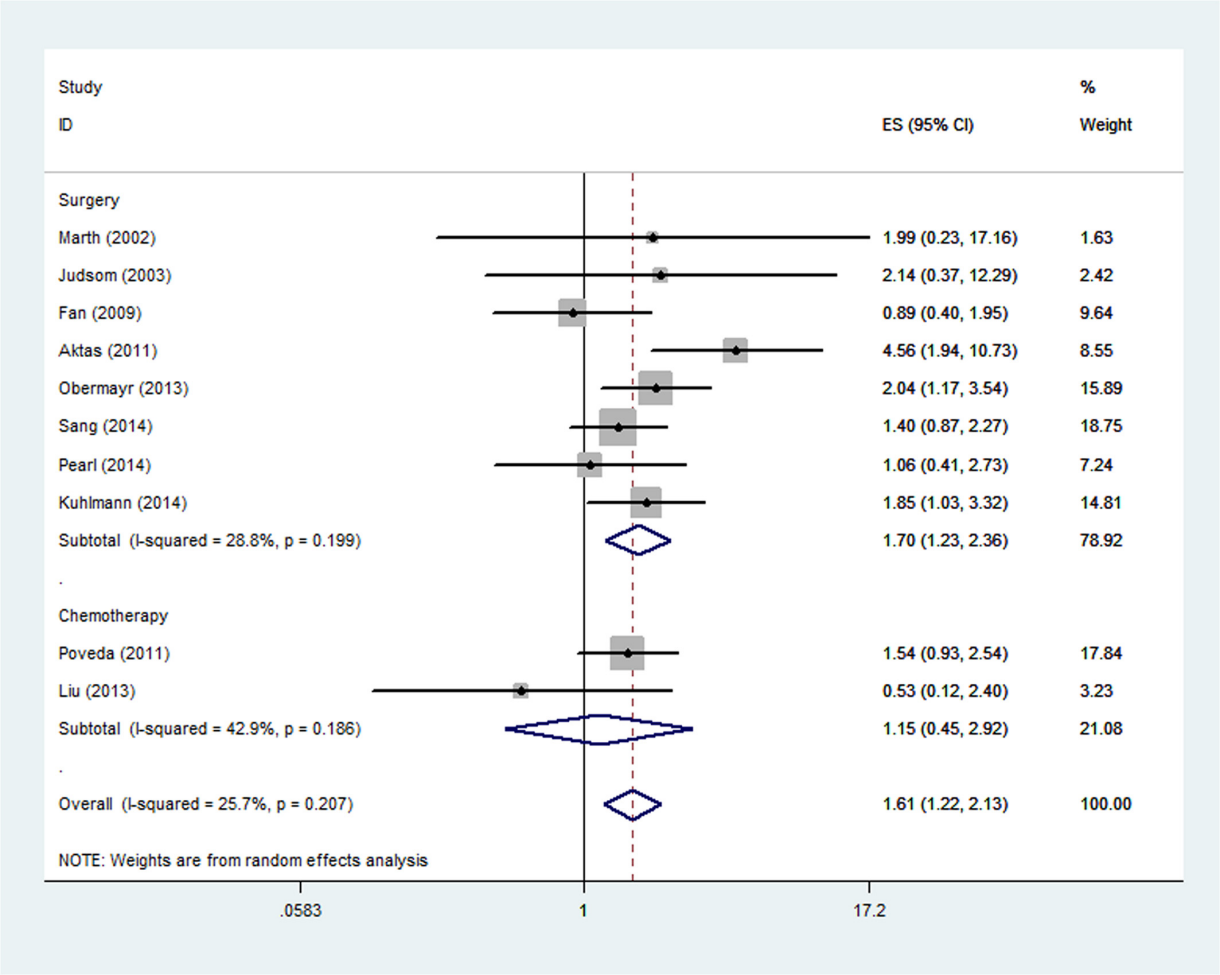
Forest plot showing the meta-analysis of hazard ratio estimates for OS in “Surgery” subgroup and “Chemotherapy” subgroup. Subgroup analysis based on different treatment methods. OS = overall survival.

When it comes to the CTC detection methods, patients present with CTC showed a significantly increased risk of mortality in the "RT-PCR" subgroup (HR, 2.02; 95% CI, 1.34–3.03; Fig 3), whereas it was not significant in the "CellSearch" subgroup (HR, 1.15; 95% CI 0.45–2.92) and "other ICC" subgroup (HR, 1.09; 95% CI 0.62–1.90). Statistical heterogeneity was not found in "RT-PCR" subgroup, "CellSearch" subgroup and "other ICC" subgroup (I^2^ = 46.7%, P = 0.131; I^2^ = 42.9%, P = 0.186 and I^2^ = 0.0%, P = 0.771, respectively). In addition, we also investigated the prognostic value of CTCs for patients who received different treatment methods. Patients received chemotherapy alone in two studies and surgery (surgery alone or surgery and chemotherapy) in the other eight studies. The results showed the prognostic value of CTCs for OS was significant in the "Surgery" subgroup (HR, 1.70; 95% CI, 1.23–2.36; Fig 4) and it was not significant in the "Chemotherapy" subgroup (HR, 1.15; 95% CI 0.45–2.92). Statistical heterogeneity was not found in both "Surgery" subgroup and "Chemotherapy" subgroup (I^2^ = 28.8%, P = 0.199 and I^2^ = 42.9%, P = 0.186, respectively).

### CTC and PFS/DFS

#### Pooled HR

**Fig 5:**
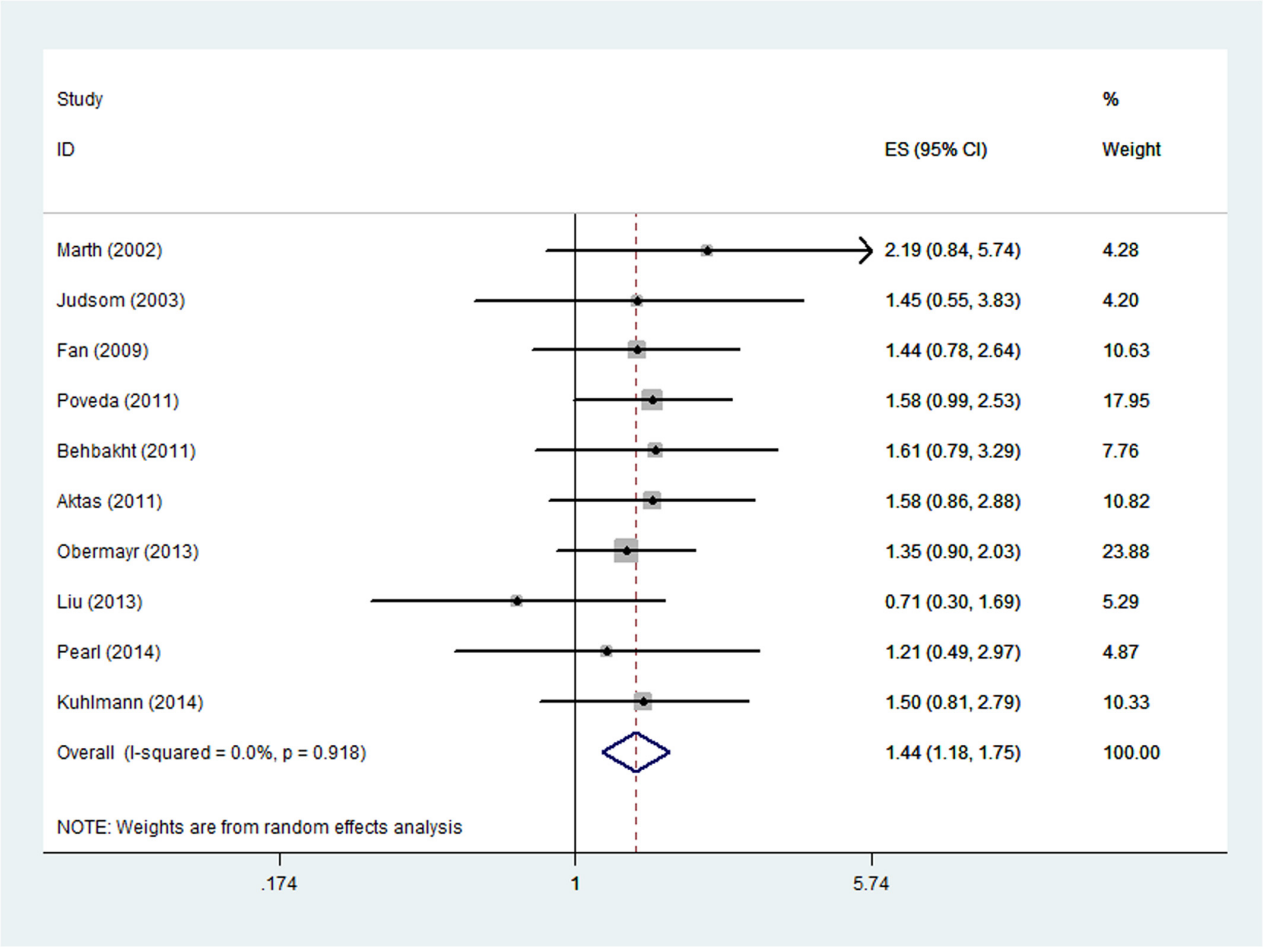
Forest plot showing the meta-analysis of hazard ratio estimates for DFS/PFS in overall patients. DFS = disease-free survival; PFS = progression-free survival.

HRs for PFS/DFS were available in ten studies [13–16,18–23]. The estimated pooled HR showed an increased risk of disease progression in patients with CTC positive group (HR, 1.44; 95%CI, 1.18–1.75; Fig 5). The heterogeneity among studies was not noted (P = 0.918, I^2^ = 0.0%).

### Meta-regression analyses

We conducted the meta-regression analysis based on the following covariates: percentage of advanced stage (stage IV or stage III), detection method (ICC vs. RT-PCR), publication year, treatment type (Surgery vs. Chemotherapy) and sample size (> = 90 vs. <90). As shown in S2 Table, no significant association could be observed in percentage of advanced stage (p = 0.645), detection method (0.118), publication year (0.619), treatment type(0.469) and sample size (0.059).

### CTC and CA-125

**Fig 6:**
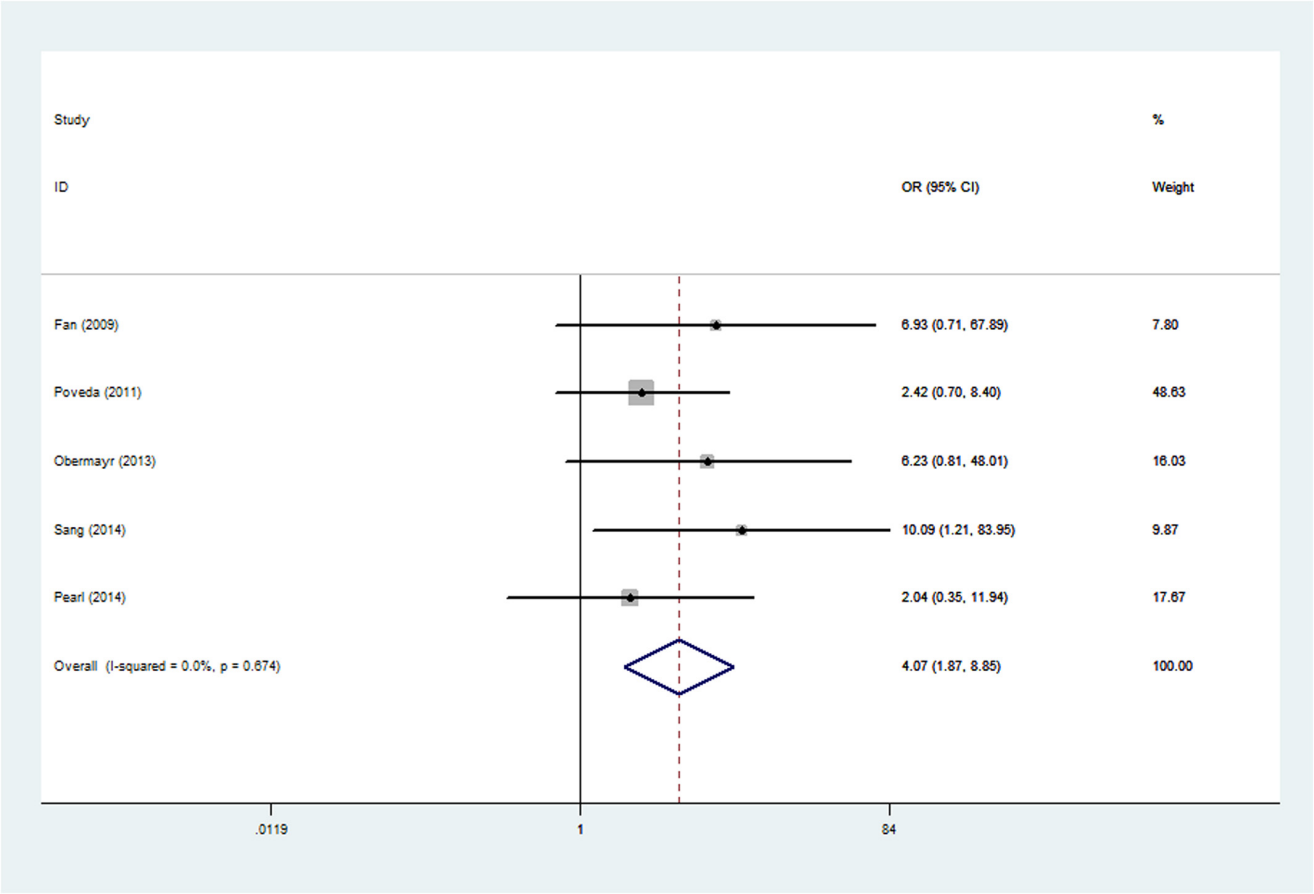
Forest plots of the association between CTCs and CA-125. CTCs = circulating tumor cells.

CA-125 is also a useful prognostic tumor marker for survival in ovarian cancer. Five studies reported data on correlation between the presence of CTC and elevated CA-125. Of these, one study [17] confirmed the correlation between CTC and CA-125. Conversely, four studies [18–20,22] suggested no correlation between CTC and CA-125. Pooled outcome from the five studies demonstrated a strong correlation between presence of CTC and increased CA-125 (OR, 4.07; 95%CI, 1.87–8.85; Fig 6).

### Sensitivity analyses and publication bias

**Fig 7:**
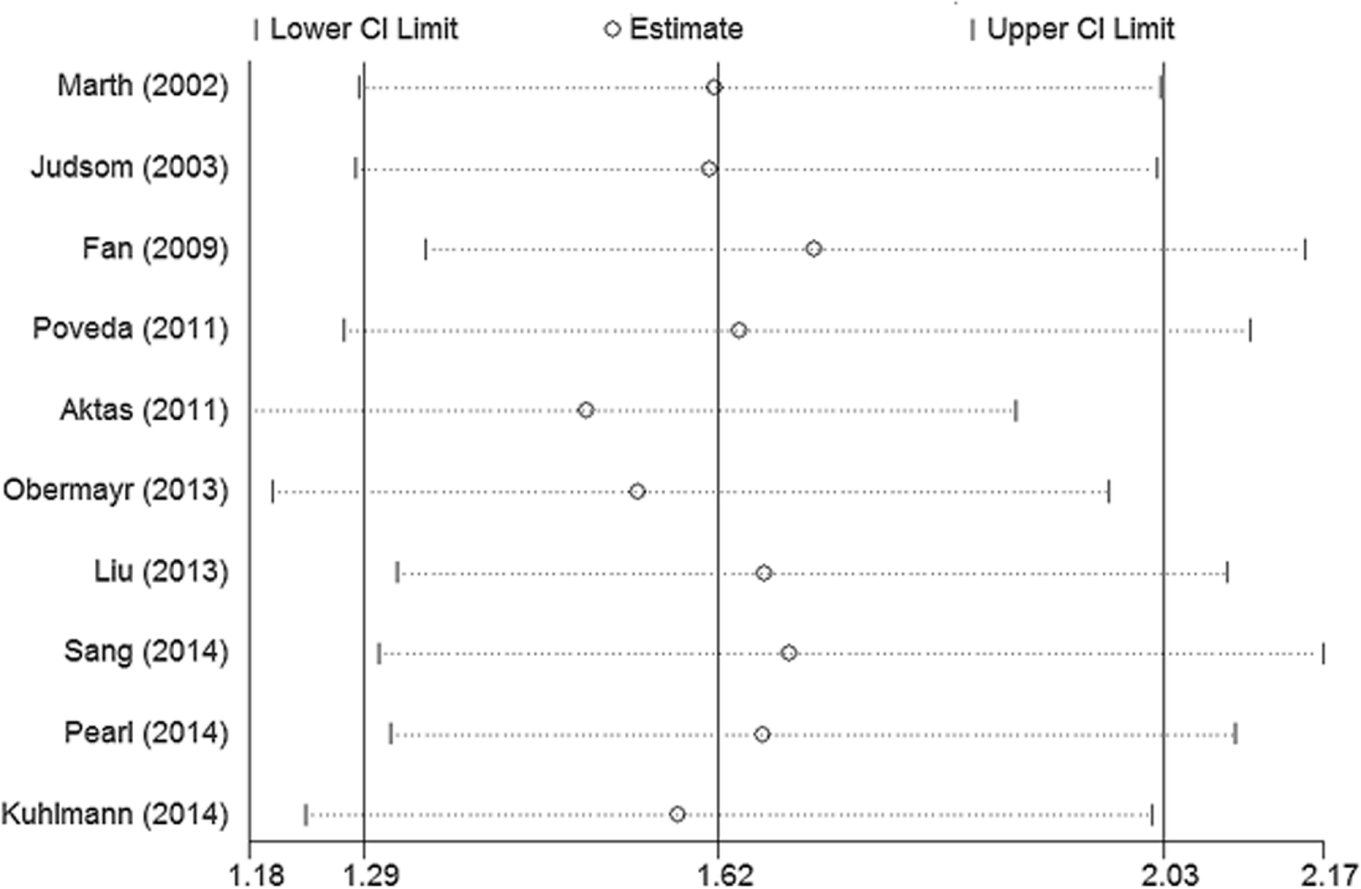
Sensitivity analysis for the pooled HRs in OS. The analysis was conducted by estimating the average HR in the absence of each study. HRs = hazard ratios; OS = overall survival.

**Fig 8:**
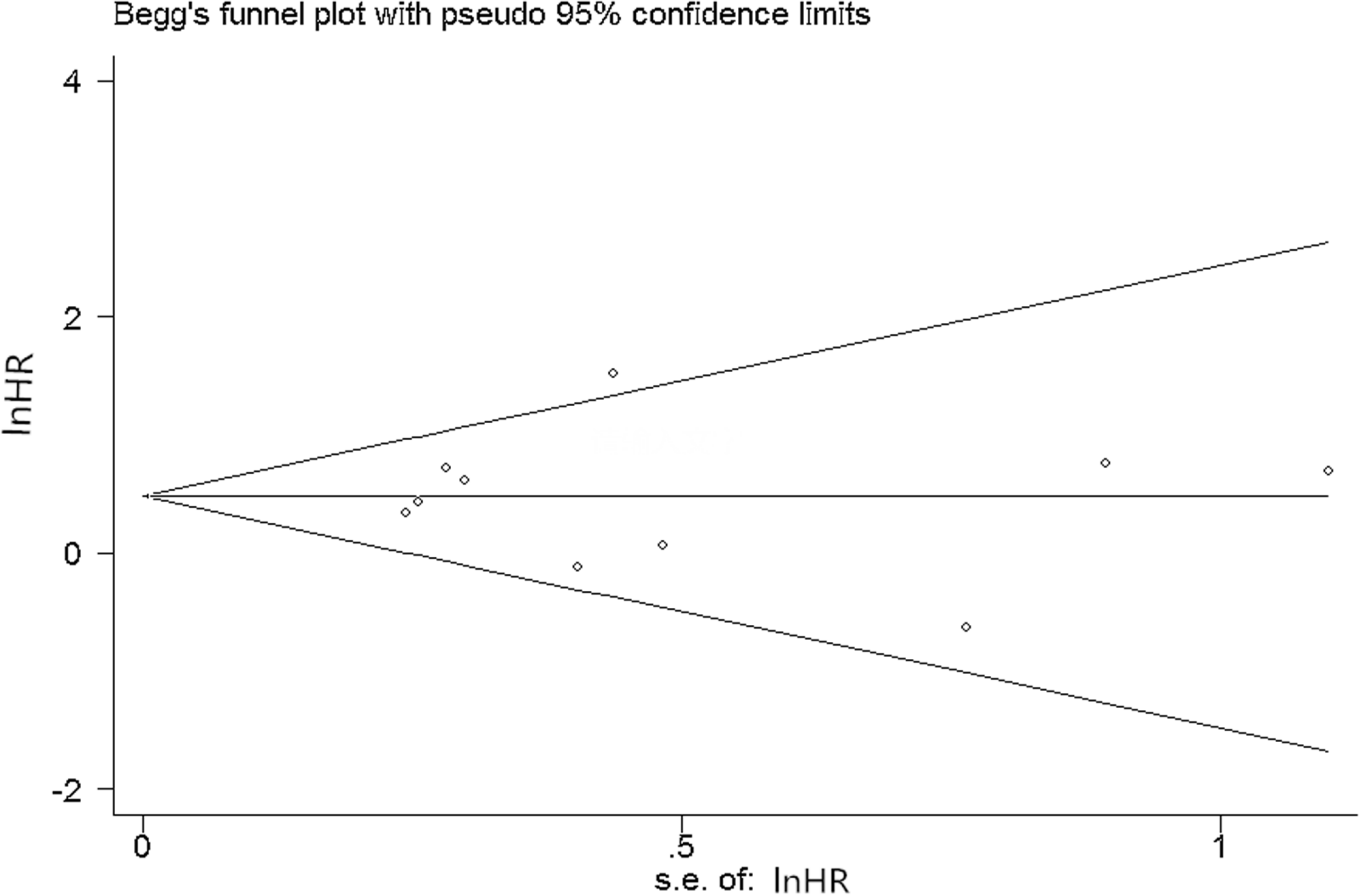
Begg’s funnel plots of the prognostic role of CTCs in OS. CTCs = circulating tumor cells; OS = overall survival.

In order to evaluate the influence of single studies on the pooled HRs, we performed a sensitivity analysis by estimating the average HR in the absence of each study. The results indicated that no individual studies significantly influenced the pooled HRs (Fig 7). Begg’s funnel plot (P = 1, Fig 8) and Egger’s linear regression test (P = 0.806) did not show evidence for publication bias.

## Discussion

Many studies have reported the prognostic value of CTCs in patients with ovarian cancer, and this is the first meta-analysis evaluating the value of CTCs in these patients. Overall, our results demonstrated that patients in CTC-positive group had a worse OS and PFS/DFS compared with CTC- negative group; moreover, the presence of CTCs was associated with elevated CA-125.

Subgroup analysis showed that “RT-PCR” subgroup presented significant association between CTCs and OS, whereas it was not significant in the "CellSearch" subgroup and “Other ICC” subgroup. But the detection method didn’t influence HR estimates in the meta-regression (P = 0.118). Though the CellSearch system which uses EpCAM for cell isolation is the only CTC test approved by US FDA for clinical use currently, it has some limitations. EpCAM can be downregulated by cancer stem cells in the procedure of epithelial mesenchymal transition [24]. Thus this system suffers from relatively low sensitivity, additional innovative detection methods may reveal more tumor cells [25]. On the other hand, CellSearch system may also present poor specificity because of biologic nonspecificity [26]. Therefore the methodologies which do not use epithelial markers alone for cell isolation may be more likely to associate with OS according to our subgroup analysis.

In addition, subgroup analysis based on treatment methods showed the prognostic value of CTCs for OS was significant in the "Surgery" subgroup, while it was not significant in the "Chemotherapy" subgroup. However, it is difficult to determine whether treatment methods influence the prognostic value of CTCs. The reason for the drawback is that there was a notable difference in timing for CTCs detection, response to treatment, chemotherapy regimens and operative plans. Further studies are required to investigate the prognostic relevant factor.

To explore potential causes of heterogeneity, we performed meta-regression to evaluate whether percentage of advanced stage patients (stage IV or stage III) would influence the prognostic value of CTCs. No association could be observed (p = 0.645), which meant the prognostic value of CTCs was not associated with disease stage. Moreover, no significant association could be observed in publication year (0.619) treatment type (0.469) and sample size (0.059).

Our meta-analysis also confirmed the presence of CTC was closely associated with elevated CA-125, both of which are known to be prognostic tool in ovarian cancer.

Our study has some limitations. First, CTC detection methods were different among included studies, which may partly influence the significance in survival analyses. Second, there is no consensus on the optimal cutoff of CTCs for predicting the clinical outcome in ovarian cancer. The low cutoff of ≥2 CTC/7.5 ml of blood with the CellSearch system was used in three studies [14,16,19], and other studies used different cutoffs. Though Fan et al. showed higher CTC counts could reflect later stage disease and higher CA-125 levels [20], few trials had evaluated by the prognostic value of different numbers of enumerating CTCs in patients with ovarian cancer. Further studies are required to assess prognostic relevant CTC cutoff levels. Third, some studies included in the meta-analysis were small case-series, thus more large prospective studies should be performed.

In conclusion, this meta-analysis indicates that CTC status is associated with OS and PFS/DFS in patients with ovarian cancer. To achieve clinical utility of CTCs in ovarian cancer, more large prospective studies should be conducted before CTC detection can be applied to clinical use as a prognostic indication.

## Supporting Information

S1 Checklist PRISMA checklist. (DOC)

S2 Checklist Meta-analysis on genetic association studies checklist. (DOC)

S1 Table The excluded articles and the reasons for exclusion. (DOC)

S2 Table Results of meta-regression analysis exploring source of heterogeneity with overall survival. (DOC)
