# Supplementary material for: Prognostic Value of Circulating Tumor Cells in Ovarian Cancer: A Meta-Analysis
Source: PLoS One. 2015 Jun 22;10(6):e0130873. doi: 10.1371/journal.pone.0130873 (PMC4476582; doi:10.1371/journal.pone.0130873)
Supplement: S1 Table — (DOC) [file pone.0130873.s003.doc]

**The excluded articles and the reasons for exclusion**

280 articles were excluded after a review of titles, keywords and abstracts because they were obviously irrelevant studies, duplicates, reviews, abstracts and comments. The following 4 articles were excluded after reviewing the full texts because of insufficient data, multiple publications and small sample size.

| Study | Reason |
| --- | --- |
| Altaras MM, Klein A, Zemer R, Zimlichman S, Bernheim J, Fishman A. (2002) Detection of tumor circulating cells by cytokeratin-20 in the blood of patients with granulosa cell tumors. Gynecol Oncol 86(3): 330-336. | Small sample size |
| Obermayr E, Alpers I, Pils D, Braicu I, Van Gorp T, et al. (2012) Circulating tumor cells in ovarian cancer: A study of the OVCAD consortium. European Surgery - Acta Chirurgica Austriaca  44 SUPPL. 248 (2) | Multiple publications |
| [Schilder RJ](http://www.ncbi.nlm.nih.gov/pubmed?term=Schilder RJ[Author]&cauthor=true&cauthor_uid=23321064), [Sill MW](http://www.ncbi.nlm.nih.gov/pubmed?term=Sill MW[Author]&cauthor=true&cauthor_uid=23321064), [Lankes HA](http://www.ncbi.nlm.nih.gov/pubmed?term=Lankes HA[Author]&cauthor=true&cauthor_uid=23321064), [Gold MA](http://www.ncbi.nlm.nih.gov/pubmed?term=Gold MA[Author]&cauthor=true&cauthor_uid=23321064), [Mannel RS](http://www.ncbi.nlm.nih.gov/pubmed?term=Mannel RS[Author]&cauthor=true&cauthor_uid=23321064), et al. (2013) A phase II evaluation of motesanib (AMG 706) in the treatment of persistent or recurrent ovarian, fallopian tube and primary peritoneal carcinomas: a Gynecologic Oncology Group study. Gynecol Oncol 129(1): 86-91. | Insufficient data |
| [Wimberger P](http://www.ncbi.nlm.nih.gov/pubmed?term=Wimberger P[Author]&cauthor=true&cauthor_uid=17764727), [Heubner M](http://www.ncbi.nlm.nih.gov/pubmed?term=Heubner M[Author]&cauthor=true&cauthor_uid=17764727), [Otterbach F](http://www.ncbi.nlm.nih.gov/pubmed?term=Otterbach F[Author]&cauthor=true&cauthor_uid=17764727), [Fehm T](http://www.ncbi.nlm.nih.gov/pubmed?term=Fehm T[Author]&cauthor=true&cauthor_uid=17764727), [Kimmig R](http://www.ncbi.nlm.nih.gov/pubmed?term=Kimmig R[Author]&cauthor=true&cauthor_uid=17764727), et al. (2007) Influence of platinum-based chemotherapy on disseminated tumor cells in blood and bone marrow of patients with ovarian cancer. Gynecol Oncol 107(2): 331-338. | Insufficient data |
