# Supplementary material for: Prognostic Value of Circulating Tumor Cells in Ovarian Cancer: A Meta-Analysis
Source: PLoS One. 2015 Jun 22;10(6):e0130873. doi: 10.1371/journal.pone.0130873 (PMC4476582; doi:10.1371/journal.pone.0130873)
Supplement: S2 Table — (DOC) [file pone.0130873.s004.doc]

| **Table S2.** Results of meta-regression analysis exploring source of heterogeneity with overall survival. | | | |
| --- | --- | --- | --- |
|  | OS  Univariate analysis | | |
| Covariates | Coefficient | SE | P |
| percentage of advanced stage | -0.356 | 0.727 | 0.645 |
| detection method | 0.473 | 0.270 | 0.118 |
| publication year | -0.169 | 0.327 | 0.619 |
| treatment type | 0.297 | 0.390 | 0.469 |
| sample size | 0.519 | 0.236 | 0.059 |
| The dependent variable is the lnHR for overall survival (OS) from each study. Weights have been assigned according to the estimated variance of lnHR. SE, standard error of the coefficient. | | | |
